# Platelet Function in Aging

**DOI:** 10.3389/fcvm.2019.00109

**Published:** 2019-08-07

**Authors:** Jessica Le Blanc, Marie Lordkipanidzé

**Affiliations:** ^1^Research Center, Montreal Heart Institute, Montreal, QC, Canada; ^2^Faculty of Pharmacy, Université de Montréal, Montreal, QC, Canada

**Keywords:** platelet function, aging, senescence, elderly, cardiovascular disease, thrombosis

## Abstract

Aging is associated with an increased incidence of cardiovascular disease and thrombosis. Platelets play a major role in maintaining hemostasis and in thrombus formation, making them a key player in thrombotic disorders. Whereas it is well-known that platelet aggregability is increased in vascular diseases, the contribution of age-related changes in platelet biology to cardiovascular risk is not well-understood. Several lines of evidence support that platelets from older subjects differ in their function and structure, making platelets more prone to activation and less sensitive to inhibition. These age-related changes could lead to platelet hyperactivity and to the development of a prothrombotic state in advanced age. This review will focus on platelet biochemical modifications during aging and on the mechanisms by which these alterations could lead to thrombotic disease.

## Introduction

Aging is accompanied by many biological changes at molecular and cellular levels. Senescence, the most striking cellular alteration in aging, is a hyporeplicative state implicated in age-related diseases (1). Senescent cells adopt a specific secretion signature rich in pro-inflammatory cytokines called the senescence-associated secretory phenotype (SASP) (1). It has been suggested that SASP induces changes in the functions of neighboring cells (2, 3), and some authors hypothesize that it might potentially contribute to increased susceptibility to thrombosis in elders (4).

Cardiovascular disease is the first cause of death worldwide and its incidence is strongly correlated with age (5). In both normal and pathological aging, platelets show increased aggregability (6–11) which is however more pronounced in a pathological state (12, 13). Several lines of evidence suggest that platelet hyperactivity observed in advanced age could be responsible for vascular and thrombotic disease development (14–17). However, molecular mechanisms of platelet hyperactivity in aging are only partially understood. A deeper understanding of the biological mechanisms underlying the relationship between age-related changes in platelet biology and cardiovascular disease could help improve treatment and preventive strategies. This review explores biochemical changes in platelets during aging.

## Age-associated decrease in Platelet Count

Platelet count is inversely associated with age. A large study based on the Third National Health and Nutrition Examination Survey including 12,142 American subjects showed a significant decrease of 10 × 10^3^ platelets/μL in individuals in the 60–69 year age group as compared with those between the ages of 20–59 years, and of 20 × 10^3^ platelets/μL in patients aged over 69 years of age, after adjusting for many covariates such as nutritional deficiencies, medication, inflammatory conditions, autoimmune or viral illnesses and consumption of alcohol and tobacco (18). This suggests that the drop in platelet count with age is part of the biological aging process *per se* and not only due to environmental factors.

Another large study on 33,258 subjects in France examined full blood count normal references values by age and sex. It showed the same tendency of platelet count being lower in older adults vs. their younger counterparts (19). Many studies showed that older women presented with higher platelet counts than men of the same age (18–20). A tendency of a lower platelet count in elderly people compared with young subjects was observed in two other small studies, but was not considered statistically significant (21, 22). Despite evidence of an inverse correlation between aging and platelet count, the cause of the decline in platelet count and the physiological consequences of this phenomenon in older subjects remain to be elucidated. Two hypotheses have been put forward in an attempt to rationalize this observation: (i) older individuals have a lower stem cell reserve compared to younger subjects; and (ii) a reduced platelet count confers a biological advantage, so the individual with this characteristic may have a better chance to reach an older age (18).

Interestingly, platelet count has been associated with mortality among elders (23–25). Van der Bom et al. reported that platelet count below 100 or above 400 × 10^9^ L^−1^ is associated with increased non-cardiovascular mortality such as cancer in a cohort of 5,766 American and African-American patients of 65 years and older (23). Another group has come to a similar conclusion, i.e., they found a U-shaped all-cause mortality curve associated with platelet count in 131, 308 Chinese patients of the same age (24). A third group confirmed the association across all ethnic groups (25). However, they found that the association between mortality and thrombocytosis or thrombocytopenia was stronger among non-Hispanic Whites compared with African-Americans or Hispanics, highlighting the effect of genetic background in age-related changes in platelet count.

Despite the decrease in platelet count during aging, reference ranges are rarely age-adjusted, leading to misdiagnosis of thrombocytopenia in the elderly. A study showed that a reference range adjusted for age and sex is more strongly predictive of all-cause mortality than the unadjusted platelet count reference range (26). This led the authors to propose that age-adjusted reference ranges might be useful in clinical practice to better identify thrombocytopenic patients with a truly increased risk of mortality (26).

## Enhanced Platelet Activity in the Elderly

One of the most documented changes in platelet function during aging is platelet hyperactivity. Bleeding time decreases significantly in aging, denoting a faster clot formation and indirectly an enhanced platelet activity in the elderly (27, 28). Furthermore, platelets from older men and women have a greater sensitivity to aggregation induced by classical agonists. Platelets aggregation of older subjects occurs at a lower concentration threshold of ADP (8–11, 22, 29), epinephrine (6, 7, 10, 11), collagen (22) and arachidonic acid (10) than platelets from younger subjects. Meade et al. reported an increase in platelet aggregability of ~8% per decade of age, calculated by the EC_50_ of ADP in a cohort of 958 participants of all ages (8). At all ages, aggregability was found to be more pronounced in women than in men (8, 10). A recent study suggests that age-related changes in platelet behavior on von Willebrand factor are more pronounced in women than in men (30), also supporting the concept that the aging process could affect platelet function differently in both sexes.

Furthermore, β-thromboglobulin and platelet factor 4 (PF4), two proteins secreted from platelets α-granules, are both found at a significantly higher level in plasma of older compared with younger subjects (29, 31). This is consistent with the hyperaggregability observed in elderly individuals since platelets release their granule content during activation. Interestingly, PF4 has also been found to have a procoagulant effect (32), showing that the age-related prothrombotic state is probably due to a number of biological changes in the thrombotic pathway, not only occurring in platelets themselves.

The mechanisms of this age-related platelet hyperactivity remain unclear. Bastyr et al. have tested the hypothesis that modifications in phosphoinositide turnover, an important signaling mechanism of platelet activation, may be responsible for platelet hyperactivity in aging (29). They have found that platelet phosphoinositide turnover is enhanced in aging and correlates positively with platelet aggregation and plasma β-thromboglobulin levels.

It has also been suggested that there could be a functional or expressional change in platelet α and β-adrenoreceptors (33), however the reported literature is conflicting. These receptors, respectively, enhanced or inhibited platelet aggregation induced by epinephrine and noradrenaline by decreasing or increasing platelet cAMP levels. Yokoyama et al. observed an increase in α-adrenoreceptor binding capacity in platelets of elderly people without change in binding affinity (7). However, two other groups have observed the opposite, i.e., a decrease in α-adrenoreceptor binding capacity (6, 34). Finally, another study suggested a decrease of β-adrenoreceptor affinity but an unchanged binding capacity in older subjects (35).

## Changes in the Platelet-Serotonin System in Advanced Age

Serotonin is a vasoactive molecule stored in platelet granules and released in plasma during platelet activation. Serotonin is also known to induce platelet shape change and boost epinephrine- and ADP-induced aggregation (33, 36). According to Gleerup et al., platelets from patients aged between 72 and 86 years old have a higher sensitivity and an increased responsiveness to serotonin than platelets from subjects 18 to 27 years of age (33). They propose that it can be an important contributing factor of increased platelet aggregability in the elderly.

On the other hand, it has been reported that platelet serotonin content decreases with age (36, 37). Diminished serotonin platelet content and an increased plasma serotonin level is also observed in patients with type 1 or type 2 diabetes and in patients suffering from peripheral vascular diseases compared with correspondingly aged healthy volunteers (36). This leads to the suggestion that platelet hyperactivity can cause an increased release of serotonin in plasma which then feeds back to platelet hyperactivity and may thus play a role in the pathogenesis of atherothrombotic diseases.

## Relationship Between Oxidative Stress, Aging, and Platelet Hyperreactivity

The human and animal aging process is accompanied by an increase in oxidative stress (38–40). Among biological systems modified by oxidative stress, the cardiovascular system is particularly sensitive to a misbalance in reactive oxygen species (ROS), which has been associated with an increased risk of cardiovascular events (38, 40). ROS also play a role in platelet activation by acting as a second messenger or by producing oxidized proteins important in platelet aggregation (38, 41), which could explain the greater propensity toward thrombosis in older individuals. Free radicals induce modifications of protein structures notably by carbonylation or cross-linking in many cell types. Those impaired proteins are therefore targeted by proteolytic enzyme as a mechanism of defense against oxidative damage. In aging, it is suggested that there is an accumulation of carbonylated proteins which could lead to the development of diseases such as diabetes, atherothrombosis and cancer. It has been observed that aging increases the rate of carbonylated proteins in platelets of rats (42), hamsters (42) and humans (42, 43), providing a biologically-plausible mechanism of platelet dysfunction. Platelet carbonyl content is even more sharply increased in type 2 diabetes when compared with healthy controls of the same age (42), further supporting the notion of ROS-induced platelet hyperactivity. Oxidative stress can also modify reactions involving disulfide bond formation, which can lead to activation or inactivation of a number of proteins implicated in different cellular pathways. For example, oxidative stress increases the activity of platelet membrane protein disulfide isomerase which activates several integrins by disulfide bond isomerization (44). The most important among these is the α_IIb_β_3_ integrin, which undergoes partial reduction of disulfide bonds during activation (44–47). Disruption of this finely-tuned balance in α_IIb_β_3_ integrin activation can thus lead to altered thrombotic susceptibility (47). This is another of many mechanisms by which oxidative stress could contribute to thrombus formation, reviewed by several authors (39, 40, 45, 48).

Nitric oxide (NO) exerts its antithrombotic effect by inducing vasodilation and by inhibiting platelet aggregation via the activation of platelet guanylate cyclase (GC) generating cGMP, which finally reduces intracellular free calcium levels (40, 49). However, NO can also contribute to oxidative stress through generation of oxidative species such as ONOO^−^, which can evolve into free radicals and cause cellular damage leading to several pathological mechanisms (50, 51). In order to investigate if age influences the platelet NO signaling system, Michimata et al. have measured GC activity in response to an exogenous NO donor in platelets of individuals of different ages (52). They found a decrease of the enzyme activity in older men (mean age 51.5 years) compared with younger men (mean age 29.5 years). Thus, the efficiency of the NO/GC/cGMP platelet pathway regulating aggregation may be modified in aging. Similarly, two other groups have examined the variation of cGMP levels in platelets with age (50, 53). Both groups showed a significant inverse correlation between cGMP platelet levels and age, suggesting a decrease in the inhibitory signaling pathway, which is consistent with Michimata's et al. results. Surprisingly, an increase in platelet nitric oxide synthase (NOS) activity in aging has been described (50), which seems contradictory with the cGMP decrease. A similar increase in platelet NOS activity and decrease in cGMP level have also been observed in 24-month-old rats, showing that this phenomenon is consistent in different species (54). Many hypotheses have been postulated to explain this contradiction, notably the possibility that the excess of NO produces higher amounts of oxidative reactive species such as ONOO^−^ which could potentially inhibit GC by targeting its iron/sulfur cluster (50).

A recent study suggests that hydrogen peroxide, an oxidative molecule, plays a key role in the platelet hyperactivity observed in aged mice (55). They found that platelets from old mice produce higher levels of H_2_O_2_ when stimulated with thrombin *in vitro* than platelets from younger mice. Furthermore, when they used PEG-catalase to selectively degrade intraplatelet H_2_O_2_ in 4- and 18-month-old mice, the authors observed the complete reversal of the age-dependent platelet hyperactivation; i.e., platelet activity of young and old mice were reduced to the same level (marked by a similar percentage of fibrinogen binding and integrin α_IIb_β_3_ activation). These findings are consistent with previous work showing that treatment with catalase inhibits platelet activation by collagen (56). The authors proposed that the increased platelet production of peroxide observed in aged mice results from enhanced NADPH oxidase/superoxide dismutase pathway. This was further supported by qPCR data showing increased NADPH oxidase regulatory subunit and superoxide dismutase-1 mRNA in platelets of 18-month-old mice. Together, these findings suggests a critical role of this oxidant molecule in platelet hyperactivity during aging.

Surprisingly, recent human and mice data suggest that beyond a certain age (~80 years for human, ~14 months for mice), intraplatelet antioxidant reserves are increased, and consequently lead to decreased reactive oxygen species platelet content and a reduction in platelet activity and apoptosis (57). The causes of this observation are still unknown, but it could be the consequences of a biological mechanism to counteract the increase of oxidative stress burden in aging.

## Age-Related Alterations in Vascular Prostaglandins

Vascular homeostasis relies on a balance between prostacyclin, which induces vasodilation and inhibits platelet activation, and thromboxane A_2_, which does the opposite (58). While thromboxane A_2_ is principally synthetized and released by platelets, prostacyclin is essentially produced by vascular endothelium.

Giani et al. have examined the sensitivity of platelets of young and old rats to exogenous prostacyclin *in vivo* (59). They found that platelets from 11-month aged rats were more sensitive to the inhibitory effect on aggregation of prostacyclin compared with 1-month old animals. They propose that this could be a natural mechanism to counteract the enhanced aggregability observed in older rats. On the other hand, Modesti et al. studied the prostacyclin receptor by performing binding assays. They found a significant decrease of expression of the prostacyclin receptor in platelets of healthy humans with age but no changes in binding affinity (60). Interestingly, a similar decrease in the number of platelet prostacyclin receptors has been observed in patients with spontaneous angina (61), suggesting that this age-related downregulation could be the beginning of an imbalance in vascular homeostasis leading eventually to a pathological phenotype.

In order to explore the effect of increasing age on thromboxane A_2_ and prostacyclin production, Reilly et al. have studied the urinary excretion of 2,3-dinor-thromboxane B_2_ and 2,3-dinor-6-keto-PGF1α, metabolites of thromboxane A_2_ and prostacyclin, respectively (27). They found that both metabolites were significantly higher in individuals aged over 50 years old than in younger individuals, suggesting that prostacyclin and thromboxane A_2_ biosynthesis is increased with age. This observation has been confirmed later by a second group who found a positive correlation between the urinary excretion of 11-dehydro-thromboxane B_2_, another stable metabolite of thromboxane A_2_, and age in 833 patients with atrial fibrillation (62). Furthermore, in this study, patients with elevated 11-dehydro-thromboxane B_2_ excretion experienced significantly more cardiovascular events during the 40 month follow-up.

## Contribution of Age-Related Plasma Membrane Modifications to Platelet Hyperactivity

The cell membrane composition is modified in the aging process, notably with a higher total amount of cholesterol, and those changes can affect diverse biochemical functions of the cell such as transcellular signalization or cell membrane ion transport (63–65). An increase in the lipid structural order, and consequently a decrease in membrane fluidity, has been shown in human lymphocyte membranes and in brain cells of mice and rats (63, 66). Cohen et al. were the first to observe similar membrane structural changes in human platelets during aging (67). Others have also seen a positive correlation between membrane structural order in human platelets, increasing age and serum cholesterol levels (67–69). An increase in lipid peroxide membrane composition, which promotes crosslink lipid-protein interactions in the membrane, has also been reported in platelets of aged rats and appears to contribute to decreased membrane fluidity (70). Interestingly, an increase in platelet membrane structural order *in vitro* is linked with a higher sensitivity to aggregation induced by ADP and epinephrine (71, 72) and a higher production of thromboxane A_2_ (73, 74). This is consistent with the increased sensitivity to aggregating agents described above.

## Platelet Transcriptome modifications in Aging

The platelet transcriptome, which includes thousands of mRNAs, miRNAs, long non-coding RNAs and other types of transcripts, has long been considered static (75). However, recent advances have rather shown that the platelet transcriptome changes dynamically in response to inflammation, cancer and other pathological states (76). Age-related modifications in the platelet transcriptome have been suggested to govern platelet function changes in aging (77, 78). RNA-sequencing of platelets from young (aged <45 years) and older individuals (aged > 64 years) revealed a total of 514 transcripts that are expressed differently in both groups (78). It is also suggested that the platelet proteome of children, adults and elders differs, but little is known about age-related changes in the expression of those proteins and their biological consequences (78, 79). Interestingly, it has been shown that the transcriptome of mouse cardiac cells varies in the presence of hypertension and in function of age, leading to variation in regulation of platelet signaling and proinflammatory mechanisms (80). This suggests that age-related changes in the transcriptome of other cell types can also modify platelet function indirectly, highlighting the complexity of transcriptome regulation in aging.

Campbell et al. have examined the changes in the platelet-monocyte relationship with increasing age (78). They observed that monocytes produce increased levels of interleukin-8 (IL-8) and monocyte chemotactic protein 1 (MCP-1) when co-incubated with platelets from older patients compared with platelets from younger patients. They suggest that granzyme A, a serine protease with increased intraplatelet expression in older patients, is responsible for excessive production of these two pro-inflammatory cytokines in monocytes. This highlights the cross-talk between platelets and immune cells as a possible mechanism by which alterations in the platelet transcriptome and proteome could contribute to inflammatory diseases in aging.

## Age-Related Hormonal Changes on Platelet Function in Women

Although men are more susceptible to suffer from cardiovascular disease than women (81), this difference of morbidity between the sexes is reduced after the age of menopause. Despite this, hormone replacement therapy (as well as hormonal contraception) does not mitigate the increased cardiovascular risk in women and may even worsen it (82–84). A number of investigators have studied the role of estrogen level changes in blood on platelet function and the link with the normal aging process in women.

Platelets and megakaryocytes express both estrogen receptors ERα and ERβ (ERβ being predominant), on which estrogen can bind and modulate gene transcription of some proteins and enzymes required in platelet function such as integrin β_3_, CD34, platelet-derived growth factor, and NOS (85). Estrogen is also known to inhibit platelet aggregation *in vitro* via the NO/GC/cGMP pathway (86, 87).

Animal studies have shown that decreased estrogen levels *per se* directly affect platelet function. According to Jayachadran et al. a surgically-induced menopause in pigs increases significantly the expression of estrogen receptors on platelets (88). In this model, they also observed an increased expression of heat shock proteins 70 and 90 in platelets, which act as ER chaperones to maintain their conformation until they bind to their ligand. An increase in NOS activity, eNOS expression and cGMP concentration have also been observed in ovariectomized pigs (88).

It has been suggested that the hormonal cycle in women of procreating age may influence platelet activity. Some authors have observed an increased platelet function during the luteal phase compared with the follicular phase and have found a negative correlation between progesterone levels and platelet reactivity (89–91), while others observed no significant differences in platelet aggregation responses during the menstrual cycle (92). Moreover, women taking oral contraception (which contains estrogen and progestogen) have greater platelet count and less spontaneous platelet aggregation than those not taking an anovulant (92). Thus, the effect of hormones on platelet reactivity is not clear.

Few well-controlled aging studies have considered the hormonal status of women as a covariate of platelet function, and therefore it is difficult to ascertain the contribution of hormonal changes on the increased thrombotic risk observed with aging (85). Indeed, when looking at platelet function in peri-menopausal women, it may be difficult to dissociate hormonal factors that vary with age from the aging process in platelets themselves. To examine the effect of menopause on platelet function with minimal impact of age, Lundberg Slingsby et al. designed a study in which they formed two groups of women: late premenopausal women and recent postmenopausal women with a mean difference age between groups of only 4 years (93). They found that platelets from postmenopausal women have a greater propensity to aggregation by classical agonists (ADP, TRAP, collagen, epinephrine) than premenopausal women. Thus, the menopause-induced drop in estrogen levels may contribute to platelet hyperactivity in women undergoing the natural aging process. Further investigations are required to fully understand the influence of estrogen and the consequences of menopause on platelet function of women, and their interaction with aging.

## Aging, Platelet Hyperactivity and Coexisting Diseases

Most studies have reported direct associations between biochemical age-related changes in platelets and their hyperaggregability. However, causality is difficult to establish in observational studies, and inverse causality has also been suggested. Kurabayashi et al. have suggested that the enhanced platelet activity observed in the elderly is caused by underlying atherosclerosis rather than by the aging process (94). They have examined shape change and peroxidase content of platelets from a small group of young and old subjects with or without atherosclerosis. They have observed no significant differences in these parameters between young and old healthy individuals. In elderly subjects with atherosclerosis on the other hand, platelets presented more frequently in an activated state (formation of pseudopods) and their peroxidase content was decreased. Although the small sample size and absence of longitudinal follow-up somewhat mitigates their conclusion, Kurabayashi et al. raise an important question by asking whether platelet hyperactivity in the elderly is caused by aging or by a coexisting onset of an atherosclerotic state. Adequately controlled longitudinal studies are thus required to answer this important question.

## Conclusion

In conclusion, aging is associated with an increase in platelet activity and a higher rate of vascular and thrombotic disease. Platelets of elderly and young humans significantly differ in terms of number, activity and structure (see Table 1). These age-related differences are closely linked to the prothrombotic state observed in the elderly population; however, the nature of this link remains only partially defined. Indeed, it is still not clear whether platelet hyperactivity and vascular disease develop concomitantly or if one is the consequence of the other. Moreover, in addition to age, many other environmental and genetic factors can influence platelet function such as elevated plasma cholesterol, tobacco and alcohol consumption, diabetes, hypertension and sexual hormones (11). Although some studies have taken into account these covariates in their analyses, it is impossible to rule out the presence of a confounding variable in such complex traits. Therefore, additional studies are required to fully elucidate the connection between molecular changes in platelets and pathophysiological changes in the vascular system during aging. A better understanding of the mechanisms underlying modifications of platelet function in aging could lead to the development of better-tailored treatments to curb thrombosis in this at-risk population.

**Table 1:** Summary of age-related changes in platelets.

	**Change during aging**	**References**
**Platelet count**	↓	(18–20)
**PLATELET ACTIVITY**		
Bleeding time	↓	(27, 28)
Platelet sensitivity to agonists	↑	(6–11, 22, 29)
β-thromboglobulin plasma level	↑	(29, 31)
PF4 plasma level	↑	(29, 31)
Platelet phosphoinositide turnover	↑	(29)
Platelet α-adrenoreceptor binding capacity	Controversial	(7) (↑), (6, 34) (↓)
Platelet β-adrenoreceptor affinity	↓	(35)
**PLATELET SEROTONIN SYSTEM**		
Platelet sensitivity to serotonin	↑	(33)
Platelet serotonin content	↓	(36, 37)
**PLATELETS AND OXIDATIVE STRESS**		
Rate of intraplatelet carbonylated proteins	↑	(42, 43)
Intraplatelet GC activity	↓	(52)
Intraplatelet cGMP levels	↓	(50, 53, 54)
Intraplatelet NOS activity	↑	(50, 54)
Intraplatelet production of H_2_O_2_	↑	(55)
**PLATELETS AND PROSTAGLANDIN PATHWAYS**		
Platelet sensitivity to prostacyclin	↑	(59)
Platelet expression of prostacyclin receptor	↓	(60)
Thromboxane A2 production	↑	(27, 62)
Prostacyclin production	↑	(27)
**PLASMA MEMBRANE MODIFICATION**		
Platelet membrane lipid structural order	↑	(67–69)
Platelet lipid peroxide membrane composition	↑	(70)

## Author Contributions

JL and ML conceived the paper. JL wrote the first draft of the manuscript. ML extensively reviewed the manuscript.

### Conflict of Interest Statement

ML has received speaker fees from Bayer for unrelated work; has participated in industry-funded trials from Idorsia for unrelated work; and has received in-kind and financial support for investigator-initiated grants from Roche Diagnostics and Aggredyne, also for unrelated work. The remaining author declares that the research was conducted in the absence of any commercial or financial relationships that could be construed as a potential conflict of interest.
